# Evaluating the Effectiveness of Suicide Prevention Gatekeeper Trainings as Part of an American Indian/Alaska Native Youth Suicide Prevention Program

**DOI:** 10.1007/s10597-023-01154-6

**Published:** 2023-08-09

**Authors:** Amelia C. Mueller-Williams, Jennifer Hopson, Sandra L. Momper

**Affiliations:** 1 Addiction Center, Department of Psychiatry, University of Michigan, Ann Arbor, USA; 2 School of Social Work, University of Michigan, Ann Arbor, USA

**Keywords:** American Indians/Alaska Natives, Suicide, Gatekeepers, Suicide prevention

## Abstract

According to the Centers for Disease Control suicide rates in 2022 for American Indian/Alaska Native youth are 2.5 times higher than the national average. An Urban Indian Health Organization’s response to this crisis was to provide community and State-wide Gatekeeper trainings between 2012 and 2019 to teach trainees (N = 810) to respond appropriately to youth at-risk of suicide. We report data on pre-, post-, and six-month follow-up surveys with trainees. Data were analyzed using generalized linear models repeated measures to test within-subject, and between-subject mean score changes on suicide prevention-related measures “knowledge,” “ask directly,” “respond,” “comfort,” and “preparedness.” Results indicated improved capacity to be prepared to address suicide in the short term and that having a graduate degree enhanced baseline suicide prevention knowledge. Over time those with less education benefited the most and better retained content. Future Trainings should engage young people and those with less education to realize the largest benefit.

## Introduction

Suicide is the third leading cause of death among youth ages 10–24 years (CDC, 2022). Suicide rates for American Indian/Alaska Native (AI/AN) youth are 2.5 times higher than the national average (CDC, 2022). Tailored programs are needed to address the suicide crisis among youth in general and for AI/ANs specifically. However, there are significant challenges to developing effective suicide prevention programming for these populations. Evidence suggests that most young people with suicidality do not seek professional help (Gillies et al., 2018; Michelmore & Hindley, 2012). This barrier to help-seeking is amplified for AI/AN youth, who often experience high levels of stigma and mistrust, and have limited access to culturally appropriate mental health care (Doria et al. 2021; Michelmore & Hindley, 2012; Tingey et al., 2014). Fortunately, most young people do seek support from informal networks such as family or friends (Kinchin et al., 2020; Michelmore & Hindley, 2012).

Effective suicide prevention relies on a community safety net, including informal helpers who recognize the signs of a person at risk of suicide and can take appropriate action (Gould & Kramer, 2001). Thus, training community members in suicide prevention skills can reduce the likelihood of a person "slipping through the cracks" while supporting the maintenance of a suicide-safer community (Wyman et al., 2008). Gatekeeper trainings are a form of education that prepares adults and youth with knowledge and skills that can be used to respond to a youth at-risk of suicide (Gould et al., 2003). These types of trainings are appropriate for a broad audience and are offered in many different settings, frequently schools and workplaces.

Research suggests that gatekeeper trainings can effectively improve suicide prevention knowledge and self-efficacy over time (Kahsay et al. 2020; Holmes et al., 2021a, 2021b). Concerns remain about how trainings can maintain improvements over the long term and promote the translation of knowledge into appropriate action (Holmes et al., 2021a, 2021b; Mo et al., 2018; Torok et al., 2019; Yonemoto et al., 2019). While gatekeeper trainings are accepted as appropriate for the general public, they are often conducted with homogenous groups, such as teachers, college students, or nurses. Research that considers individual characteristics suggests that professional role can impact the efficacy of trainings, though the effect may depend on the depth of training (Burnette et al., 2015; Condron et al., 2019; Cross et al., 2011; Lamis et al. 2016). Beyond professional role, scant research has been done to consider the role of other individual factors including educational attainment on the uptake and retention of suicide prevention knowledge. This may be especially important as gatekeeper trainings vary in terms of in-depth nature and teaching style.

Individuals in youth-facing roles such as social workers, healthcare providers, and teachers have high exposure to youth and thus are in positions to identify and refer at-risk youth as needed. However, these professionals lack adequate and consistent training on suicide prevention in their formal training and continuing education training (Kahsay et al., 2020; Kreuze et al., 2018; Schmitz et al., 2012; Sylvara, & Mandracchia, 2019). Gatekeeper trainings are a good option to fill this gap, but more knowledge is needed to guide recommendations on the best training type across professional roles and settings.

For suicide prevention among AI/AN youth, culturally specific education is needed to improve the efficacy of gatekeeper trainings for this population (Cwik et al., 2016; Hopson et al., 2022; Nasir et al., 2016; Pham et al., 2021). As 71% of AI/ANs live in metropolitan areas, the youth population can be difficult to access for tailored prevention efforts outside of tribal settings (Urban Indian Health Institute, 2022). Additionally, those who identify as AI/AN are frequently mis-identified as another race, including on official documents (Arias et al., 2016; National Council of Urban Indian Health, 2019). Thus, in many settings individuals may be interacting with AI/AN youth without knowing it and may miss opportunities to engage in appropriate suicide prevention efforts with this high-risk group.

In response to the identified need for youth suicide prevention in AI/AN communities, the “Manidookewigashkibjigan Sacred Bundle: R.E.S.P.E.C.T. Project” was established as a community-based participatory research collaboration between American Indian Health and Family Services (AIHFS), an Urban Indian Health Organization, and the University of Michigan from 2011–2019. The project aimed to develop youth suicide prevention and wellness promotion programs in the seven-county area around Detroit in Southeast Michigan and in Tribal settings across the state. An integral program developed as part of this project was culturally informed gatekeeper training programs provided for community members and service providers that may interact with AI/AN youth, including students and youth themselves.

This study reports on pre-, post-, and six-month follow-up survey data collected from individuals trained in one of two types of gatekeeper trainings, Applied Suicide Intervention Skills Training (ASIST) or Suicide Alertness for Everyone (SafeTALK). Both trainings used are commonly used evidence-based suicide prevention gatekeeper trainings developed by the organization LivingWorks, a world leader in suicide intervention training. Gatekeeper trainings are commonly completed among homogenous groups of participants, such as school workers or nurses. Because of this project’s community-based approach, a diverse group of individuals participated in trainings, including across educational background. Here we evaluate the trainings’ short- and longer-term effectiveness to elicit self-rated knowledge, preparedness, and behavioral intentions to address suicide among youth, and how training effectiveness may depend upon participants’ education status.

## Methods

Between 2012–2019, 56 suicide prevention gatekeeper trainings were conducted including ASIST (*n* = 23 TRAININGS) and SafeTALK (*n* = 23) at the AIHFS facility, Tribal locations, public colleges, and community organizations in Michigan. ASIST is an in-depth two-day training including interactive practice of suicide intervention skills; SafeTALK is a shorter, four-hour training that focuses on identifying individuals at risk and connecting them to help. Livingworks worked with our team to create modified trainings with vignettes featuring AI/AN characters and incorporating AI/AN cultural norms.

SafeTALK and ASIST trainers had a range of backgrounds and lived experiences; most were AIHFS staff members. Trainers attended a “Training for Trainers” session and were required to co-teach three trainings before becoming fully certified. The majority of trainings included at least one trainer who identified as AI/AN, those who were not AI/AN were allies with extensive experience working in AI/AN communities. In cases when an AI/AN trainer was not present, an AI/AN person served as a “helper” to provide cultural guidance and foster trust.

Training participants were recruited using flyers, online announcements, and in-person at the AIHFS clinic and through partnering Tribes, universities, and behavioral health organizations. Training enrollment was open to all those interested, ages 16 years and older for ASIST and 15 years and older for SafeTALK. Recruitment focused on those most likely to interact with youth or the AI/AN community. Thus, almost a third of the trainings were held in tribal settings. This study was approved by the Institutional Review Board at the University of Michigan as not regulated.

### Procedures

The research design implemented surveys as pre-tests (t1) immediately before the training began, post-tests (t2) immediately after, and six-months post training (t3). Six-month follow-up was conducted using a database of participants’ contact information compiled from sign-in sheets. Participants were contacted up to six times to complete the t3 survey and were able to complete the survey online or over the phone for a $20 incentive gift card. Response rates to surveys were similar across time points for both trainings. Post-test surveys (t2) were completed by 97% of participants, and 6-month follow-up surveys (t3) were completed by 55% of participants.

### Participants

The demographic characteristics for both trainings were comparable (Table 1). For the entire sample, the average age was 38.5 years, most participants identified as women, and most were non-Hispanic White or AI/AN. About half of the participants had a bachelor’s degree or less. Since the trainings focused on training gatekeepers with high likelihood of exposure to youth, some recruitment occurred on college campuses, including those training clinicians such as social work and nursing; thus about 39% of the participants with less than a bachelor’s degree were students.

**Table 1:** Gatekeeper training participant demographic characteristics by training type

Characteristics	% ASIST (N = 404)		% SafeTALK (N = 405)	
*Gender*				
Woman		79		79
Man		19		18
Other		2		3
*Race/Ethnicity*				
AI/AN alone or in combination		20		22
Asian/Pacific Islander		1		3
Black		11		5
White		56		54
Hispanic/Latinx		2		7
Other		5		4
Missing		5		5
*Education group*				
< Bachelor’s degree		28		29
Bachelor’s degree		23		21
Graduate student		12		12
Graduate degree		36		36
Missing		1		2

### Instrumentation

Surveys included demographic information and evaluated participants’ self-rated thoughts, knowledge, and experiences in several suicide prevention related areas. The five key indicators evaluated were knowledge, behavioral intention, ability, comfort, and preparedness in responding to an individual at risk for suicide. Data on knowledge came from a mean composite score in response to three suicide prevention knowledge questions on a 5-point scales (1 = strongly disagree, 5 = strongly agree): “It is appropriate to ask someone who may be at risk of suicide about suicide;” “I know how to get help for someone who is at risk of suicide;” and “I can identify warning signs and risk factors for suicide.” Data on the other factors came from participant responses to the following statements on a five-point scale: “If someone appears to be at risk of suicide, I will ask them directly if they are thinking of suicide” (1 = strongly disagree, 5 = strongly agree, “ask directly”); “I can respond to suicidal behavior” (1 = strongly disagree, 5 = strongly agree, “respond”); “Indicate your comfort level with discussing suicide with others” (1 = not at all comfortable, 5 = very comfortable, “comfort”); “Indicate your preparation level with responding to a youth who is exhibiting depressed and/or suicidal behavior” (1 = not at all prepared, 5 = very prepared, “preparedness”).

### Data Analysis

Data were analyzed using GLM repeated measures to test within-subject, and between-subject mean score changes on the suicide prevention-related measures “knowledge,” “ask directly,” “respond,” “comfort,” and “preparedness.” Mean scores were tested between two time point dyads t1-t2 (nASIST = 404, nSafeTALK = 377) and across all three time points t1-t2-t3 (nASIST = 233, nSafeTALK = 212). Education level grouping of less than a bachelor’s degree, bachelor’s degree, graduate student, or graduate degree was entered as a between-subjects factor when modeling t1-t2-t3 to compare the longitudinal training effects for participants with different educational backgrounds. T-tests were used directly compare the mean scores for each measure and each education level (two-sided *p* < 0.05) from ASIST and SafeTALK, differences in mean scores between the two trainings and 95% CIs are reported (negative score differences indicate higher scores from SafeTALK).

## Results

**Fig. 1:**
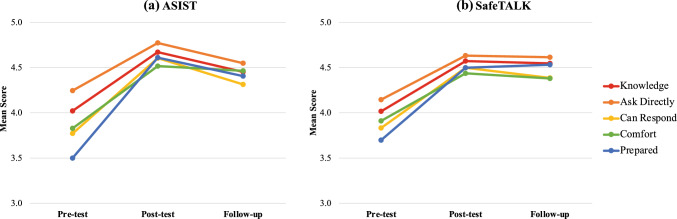
Mean scores on suicide prevention training measures across time points

The main effect of time was significant (*p* < 0.05) for both trainings across all measures. Figure 1 depicts the trajectory of mean scores over the three time points for all measures by training type. For both ASIST and SafeTALK, the mean scores improved significantly from t1-t2 (*p* < 0.05) on all measures demonstrating excellent uptake of information. Scores at t2 were significantly maintained at t3 on all measures for SafeTALK trainees but only on “comfort” for ASIST, indicating greater long-term retention for SafeTALK than for ASIST.

**Fig. 2:**
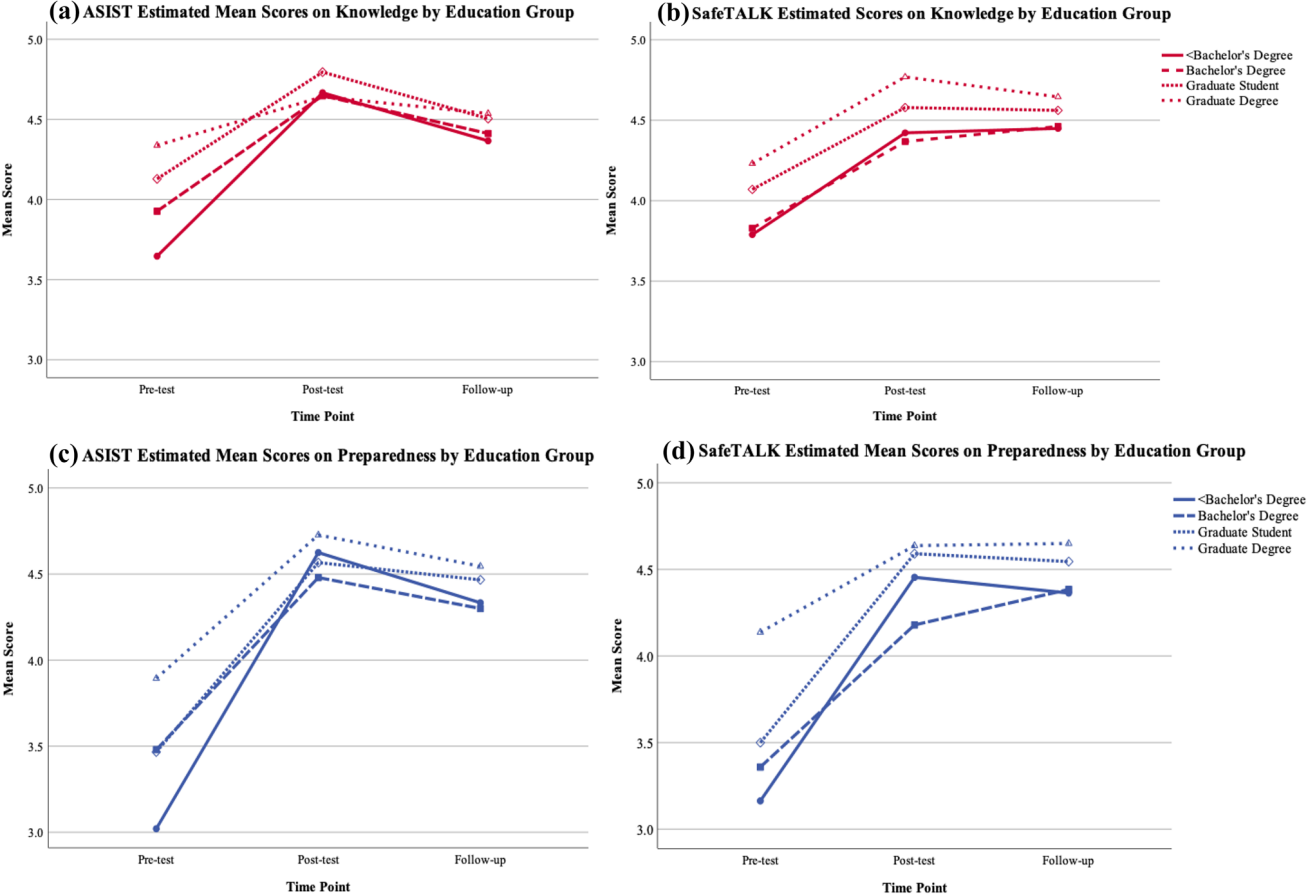
Estimated mean scores on suicide prevention knowledge and preparedness to address suicide risk among youth by education group and training type

When considering education group in the analysis, the main effect of time was also significant on all measures for both trainings (*p* < 0.05). The between education group effect was also significant (*p* < 0.05) for all measures on both trainings except for ASIST on “ask directly.” The interaction of education group and time was significant for both trainings on “preparedness,” “knowledge,” and “respond” for ASIST. Figure 2 depicts the trajectory of mean scores over the three time points by education group and training type for measures of “knowledge” and “preparedness.”

Generally, for SafeTALK, higher education levels tended to score higher; this trend is observed for all measures except “preparedness.” For ASIST this pattern was not consistent, though those with graduate degrees scored highest overall on most measures. However, those with less education showed the most improvement. Both trainings were the most effective at increasing and maintaining scores for those with a bachelor's degree or with less than a bachelor's degree. There were significant t1-t2 score improvements (*p* < 0.05) for the two lowest education groups for both trainings on all measures. Increases were maintained except for ASIST trainees with a bachelor’s degree on “ask directly.”

The two highest education groups (graduate students and those with a graduate degree) generally had higher scores at t1 but did not consistently improve t1-t2 or maintain improvements at t3. The graduate student SafeTALK trainees showed less improvement than other groups with no significant score improvement between t1-t2 except on “preparedness.” In contrast, graduate student ASIST trainees showed a significant t1-t2 score improvement on all measures, but it was not maintained at t3 on measures of “knowledge” and “ask directly.” Among those with a graduate degree, there was no significant increase in scores for ASIST trainees on “knowledge” or “ask directly,” and significant score increases were only maintained from t2-t3 for “comfort” (*p* < 0.05). For SafeTALK graduate-degree-level trainees there was significant improvement on scores for all measures (*p* < 0.05), but the improvements were not maintained on “ask directly.”

For direct comparisons of mean scores between ASIST and SafeTALK using all participants, there were significant differences (*p* < 0.05) at t1 on “ask directly” (*M*_difference_ = 0.15, 95% CI: 0.033, 0.26), at t2 on “preparedness” (*M*_difference_ = 0.17, 95% CI: 0.086, 0.253) and “comfort” (*M*_difference_ = 0.11, 95% CI: 0.02, 0.20), and at t3 on “knowledge” (*M*_difference_ = − 0.0946 (− 0.19, − 0.0004). Among those with less than a bachelor’s degree, there were significant differences (*p* < 0.05) between ASIST and SafeTALK participants’ mean scores on “comfort” at t1 (*M*_difference_ = 0.26, 95%CI: 0.034, 0.50) and t2 (*M*_difference_ = 0.22 (0.054, 0.40). For those with a bachelor’s degree, there were significant differences (*p* < 0.05) in mean scores at t1 on “ask directly” (*M*_difference_ = 0.122 (95%CI: 0.046, 0.53), and “knowledge” (*M*_difference_ = 0.211 (95%CI: 0.014, 0.41) and for the same measures at t2 (“ask directly” *M*_difference_ = 0.26 (95%CI: 0.060, 0.50); “knowledge” *M*_difference_ = 0.21 (95%CI: 0.012, 0.40). Mean scores were also different (*p* < 0.05) at t2 on “comfort” (*M*_difference_ = 0.24 (95%CI: 0.052, 0.43) for bachelor’s level participants. Among graduate students, mean scores were significantly different (*p* < 0.05) between the two trainings at t2 on “comfort” (*M*_difference_ = 0.289 (95%CI: 0.080, 0.501) and “preparedness” (*M*_difference_ = 0.22 (95%CI: 0.010, 0.44). Finally, among those with a graduate degree, the significant differences (*p* < 0.05) in mean scores between ASIST and SafeTALK occurred at t2 for “ask directly” (*M*_difference_ = − 0.17 (95%CI: − 0.31, − 0.031) and “knowledge” (*M*_difference_ = − 0.15 (95%CI: − 0.27, − 0.018). At t3 there were no significant differences on any measure for any education group.

## Discussion

Overall, gatekeeper trainings seem to improve individual-level reported capacity to address youth suicide, at least in the short-term, for both trainings and longitudinally for SafeTALK and on “comfort” for ASIST. The largest observed effect of the training was on “preparedness,” especially from t1-t2. This finding is logically expected as the construct “preparedness” incorporates multiple different aspects of suicide prevention training, so it may include variation in individuals’ feelings related to the other variables. For example, maybe one feels less intention to “ask directly” about suicide but feels that the training, overall, significantly increased their “preparedness” to address suicide through other means such as increasing knowledge.

The significant interaction between time and education group on most measures for ASIST, and “preparedness” for SafeTALK suggests that trainees with different educational backgrounds demonstrate different patterns of knowledge, uptake, and retention from these suicide prevention trainings. However, this differential response is generally present for ASIST but not SafeTALK. Trends in participant scores tended to be parallel over time for SafeTALK but not for ASIST, suggesting education level may influence performance at a similar magnitude for SafeTALK but not ASIST. This finding could be explained by the difference in training depth between the two: ASIST is a more intensive training that included active role play and a focus on cultural competency for AI/AN communities as part of examples and scenarios. People who have less education or who are not working in interactive youth-facing environments may be less comfortable with this type of in-depth activity, which could explain the difference in response across education groups for ASIST trainees. This is consistent with previous research demonstrating that skill practice during gatekeeper trainings does not significantly improve training outcomes over time, though it does have a beneficial effect in the shorter term (Cross et al., 2011). Thus, more basic, and shorter training types such as SafeTALK may be sufficient at least as an introduction to youth suicide prevention and more accessible to a broader audience.

Findings from the direct comparison of mean scores between ASIST and SafeTALK within education groups and across time periods are consistent with those from the GLMs. While scores among ASIST participants tended to be higher, as did those of participants with higher education, there was no significant difference in mean scores for any education group after six months. This suggests that despite being much less in-depth, SafeTALK performed just as well as ASIST in scores after six months for all participants.

Among all participants, the general trend indicates that those with a graduate degree have consistently higher scores. About two-thirds of the graduate-degree level trainees worked in behavioral health or child welfare services, thus it is reasonably expected that their baseline suicide prevention skills would be higher. Anecdotally, participants with a graduate degree (particularly in social work and psychology) commented that they did not find the SafeTALK training to be informative or provide new information. This is consistent with previous research that reports those in health-related roles or those requiring higher education (e.g., social workers) have higher baseline suicide prevention knowledge (Lamis et al. 2016; Smith et al., 2014; Cross et al., 2011).

This study finds that those in the lower education grouping may benefit the most and better retain training content over time. Previous research has almost exclusively focused on training outcome variation by professional role, and results are mixed. For example, in a study of Veteran’s Administration clinical and non-clinical staff both improved pre- and post-training, but the non-clinical staff had larger gains (Matthieu et al., 2008). However, another study found that higher-level professional roles in school settings, including social workers and health professionals, responded best to in-depth trainings (Condron et al., 2019). Another study from the Netherlands found trainees in professional roles had similar training outcomes, but healthcare professionals scored highest overall (Terpstra et al., 2018). Lamis et al. (2017) found that school guidance counselors scored highest on suicide prevention at baseline, but had the lowest gains compared to other roles. Together with the present findings, this may suggest that education group is not a direct proxy for professional role, and individual education background may exert a different effect on suicide prevention training efficacy. However, the efficacy of training in terms of improvements from baseline may depend on participants’ starting knowledge or experience in the area.

### Limitations

Though response rates were reasonably robust, there was significant loss to follow-up at six months. The individuals who responded to follow-up may not be representative of the entire sample of trainees or reflect bias based on individual tendency to respond. Additionally, all measures were self-reported. These measures do not necessarily translate into individual’s actions related to youth suicide prevention. Other factors, such as age, gender, or type of employment may influence individual responses to the gatekeeper trainings. For example, there may be a high level of age-related factors influencing the outcomes among those with less than a bachelor’s degree because the group is made up of both young students and older people. Though each training included an AIAN leader, in some cases, these individuals were not established members of the community in which the training took place. Thus, the uptake and retention of training material could have been impacted by the level of trust fostered by trainers and groups of participants.

## Conclusion

Continued community-based engagement in suicide prevention is critical as suicide rates are increasing nationally and contribute to decreasing and stagnating life expectancies across the nation (National Academies of Sciences, Engineering and Medicine 2021). This study supports the use of gatekeeper trainings as one part of community-based suicide prevention programs but suggests that trainings should be tailored to specific audiences in order to gain maximum benefit. Findings suggest that despite differences in short-term gains, after six months, the skills from these two trainings are not different for participants of all educational backgrounds. For individuals with less education or suicide prevention experience, more training may not be best, at least at first; it may be beneficial to start with introductory trainings (e.g., SafeTALK) before proceeding to more in-depth training (e.g., ASIST). The integration of culturally responsive approaches may be used to meaningfully engage participants working in AI/AN communities, but this may not be feasible for the shorter SafeTALK training. A potential approach could include providing an AI/AN-specific training booster training after six months, such as “Gathering of Native Americans” (GONA) or Native H.O.P.E. (“Helping our People Endure”).

Future research should evaluate whether mix-and-match approaches to suicide prevention education are more effective for retention of skills and confidence, and whether ASIST is more effective when participants have had some earlier exposure to suicide prevention. We suggest that future gatekeeper trainings should focus on engaging young people and those with less education in order to promote the largest benefit for the community. For targeting the AI/AN community, research and practice should incorporate culturally relevant approaches and evaluate the efficacy of those approaches on AI/AN-specific engagement.
